# Resolving individual atoms of protein complex by cryo-electron microscopy

**DOI:** 10.1038/s41422-020-00432-2

**Published:** 2020-11-02

**Authors:** Kaiming Zhang, Grigore D. Pintilie, Shanshan Li, Michael F. Schmid, Wah Chiu

**Affiliations:** 1 Department of Bioengineering and James H. Clark Center, Stanford University, Stanford, CA 94305 USA; 2 Division of CryoEM and Bioimaging, SSRL, SLAC National Accelerator Laboratory, Menlo Park, CA 94025 USA

**Keywords:** Cryoelectron microscopy, Molecular modelling

Dear Editor,

Breakthroughs in single-particle cryo-electron microscopy (cryo-EM) technology have made near-atomic resolution structure determination possible. Cryo-EM has resolved over four thousand structures at near-atomic resolutions (2–4 Å).^1^ It is rapidly becoming the method of choice for structure determination of membrane proteins, large assemblies, and multi-protein complexes partly because it does not require a crystal and partly because it can accommodate specimens with heterogeneous composition and/or conformation.^2^ This powerful technique is now capable of resolving protein complexes to better than 2 Å resolution^3^ and has been used to solve 3.7 Å resolution structure of RNA as small as ~40 kilodaltons.^4^

Two years ago, we obtained a 1.75 Å resolution apoferritin structure^3^ using ~70,000 particle images through 10 h of data collection using a Gatan K2 detector in a Titan Krios G3i electron microscope. Recently the K3 and Falcon 4 detectors were installed on two of our Titan Krios G3i electron microscopes at the Stanford-SLAC Cryo-EM Center, providing us with three times the data collection rate. In this study, using the same apoferritin, we first collected a new dataset using the K3 detector to find out what is the highest resolution structure achievable. The dataset was collected in the electron counting mode using “Faster Acquisition” in EPU, with a throughput of ~520 movie stacks per hour and a data acquisition result of 8000 movie stacks per 16 h (Supplementary information, Table S1). Using a standard image processing pipeline (Supplementary information, Fig. S1a),^5^ a density map of apoferritin with a resolution of 1.34 Å was obtained from ~900,000 particles (Fig. 1a–d). In addition, we also collected a second dataset using a Falcon 4 detector in the electron counting mode (MRC format, not EER format) with a throughput of ~500 movie stacks per hour, resulting in ~7700 movie stacks from a 15 h data collection. A 1.36 Å resolution map of apoferritin was obtained from ~500,000 particles (Supplementary information, Fig. S1b–e and Table S1). Both maps have comparable resolution based on the Fourier Shell correlation of 0.143 threshold^6^ and similar Gaussian “B-factor”, relating the falloff in resolution of the reconstructions to the numbers of particles^7^ (Supplementary information, Fig. S1f, g). This “B-factor” indicates the quality of the map, which is caused by a combination of the experimental limitations of the specimen and the instrument, and the errors inherent in the computational image processing pipeline. In comparing the atom positions between the two independently optimized models from these maps, they were found to have a root mean square deviation (RMSD) of 0.27 Å for all atoms, 0.07 Å for backbone atoms, and 0.41 Å for side chain atoms. These results validate the reproducibility and precision of the maps obtained from two independent data sets and instruments.

**Fig. 1:**
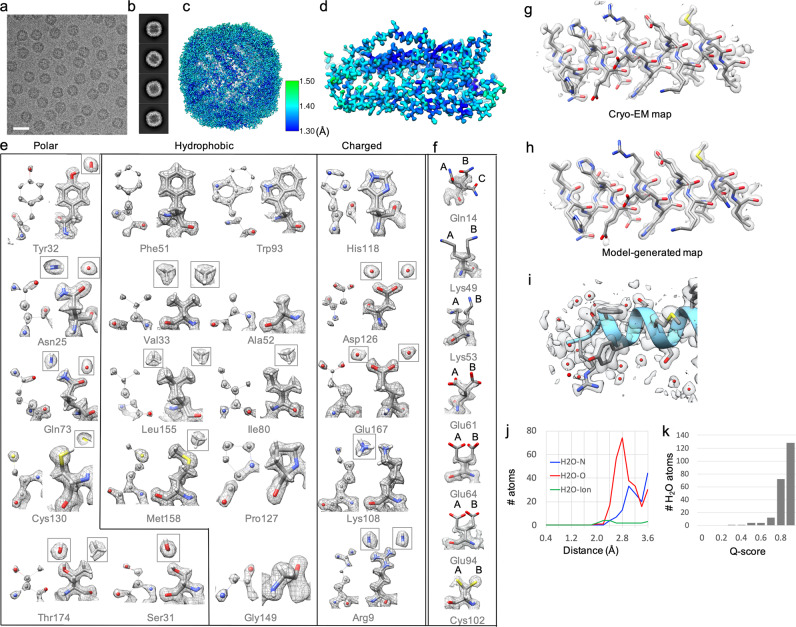
Atomic resolution structure of apoferritin determined from a 300 kV Titan Krios G3i electron microscope with K3 detector. **a** Representative motion-corrected cryo-EM micrographs. The scale bar represents 200 Å. **b** Reference-free 2D class averages of computationally extracted particles. **c** Resolution variation maps for the final 3D reconstruction. **d** Cryo-EM density map of an extracted single subunit. **e** Twenty representative amino acids extracted from the 1.34 Å resolution map. The amino acids were selected based on the type of side chain (polar, charged, and hydrophobic). Each residue is shown on a higher density display level (0.045 in Chimera, left) or lower density display level (0.008 in Chimera, right), showing separable/resolved atoms or shapes of atoms including hydrogen atoms, respectively. **f** Representative residues with alternate conformations of side chains. A/B/C represents different side-chain conformations. The residues in **e** and **f** are shown by elements (grey, carbon; red, oxygen; blue, nitrogen; yellow, sulfur; white, hydrogen). **g**, **h** A representative helix was extracted from the cryo-EM density map (**g**) and the model-generated map using B’ factors (**h**). **i** Water molecules are shown around a small portion of the helix. **j** A radial distance plot between water and O atoms in the protein shows a sharp peak at 2.8 Å resolution. **k** A histogram of Q-scores for placed waters shows that most are placed in well-resolved peaks with Q-scores of 0.8 and higher.

Our maps show separable atom densities at a properly chosen contour level, well-resolved side-chain atom densities, and even some indications of hydrogen densities as exemplified from the 1.34 Å resolution map (Fig. 1e). The presence and direction of the density for hydrogen, even though they are not distinctly resolved, are visually compelling. Furthermore, the difference between the oxygen (O) and the nitrogen (N) atoms in Asparagine (Asn) and Glutamine (Gln) residue side chains, where the N has extended density for its hydrogen (H) atoms, whereas the O appears circular in an end-on view, allows one to distinguish these two atoms, which informs whether the atom is a hydrogen bond donor or acceptor. The same is the case for the O vs. C atoms in Threonine (Thr), wherein the terminal methyl group is roughly triangular in shape, and the O again appears circular in an end-on view, and allows one to unambiguously differentiate Thr from Valine (Val). However, the side chains of ~12 residues cannot be clearly identified in either map due to multiple rotameric conformations (a few examples shown in Fig. 1f), which may be caused by their inherent or electron radiation-induced dynamic properties.

The Molprobity and PDB reports of our two models rank very highly in all the assessment scores on the adherence of models to the chemical properties of proteins (Supplementary information, Fig. S2). Generally speaking, it is difficult to assess the fit of a model to a density map by visual display, partly because of the choice of contour display and partly because of the variation of resolvability throughout the map (Fig. 1c, d; Supplementary information, Fig. S1e). Though the overall resolutions of maps are reported based on the Fourier shell correlation (Supplementary information, Fig. S1f), some side chains and residues are less resolved. We used Q-scores, a recently proposed cryo-EM structure validation method, to measure the resolvability of individual atoms.^3^ Q-scores were shown to correlate strongly to the resolution of the map, with the best score normalized to 1.0. Supplementary information, Fig. S3a shows the per-residue Q-score plot for our two maps range between 0.85 and 0.88; most residues have average Q-scores at or above the expected level for this resolution.^3^ However, a few dips in the plot can be seen, indicating lower average Q-scores at turns or loops between helices. Visual inspection of the residues with lower Q-scores confirms that the side chains are less resolved for these residues. Such residues tend to have alternate conformers (Fig. 1f) and be on the exterior surface of the complex.

Traditionally, individual B-factor of atoms used in X-ray crystallography is used to assess the atom position uncertainty in crystallography and is a weighting factor to allow computing model-based structural factors identical to the observed structural factors. Note that the crystallographic B-factors are not the same as the “B-factor” as described above although both have the same mathematical representation of a Gaussian function for describing the speed of the falloff in Fourier space. The crystallographic B-factors are estimated by iterative and simultaneous refinements of agreement in Fourier amplitudes between observed and computed values, and Fourier phase estimation. In cryo-EM, the modeling software yields crystallographic equivalent B-factors also known as atomic displacement parameters. However, these parameters generally are not optimized to match a model-based map with the experimental map.^8^ We thus introduce B’ factors derived from per-atom Q-scores; the calculation involves a simple scale factor determined empirically by testing which value makes the resulting model-derived map match the cryo-EM map better by Fourier Shell Correlation (Supplementary information, Fig. S3b–e). The B’ factors for each atom, which will be deposited to the PDB along with their coordinates, serve the same purpose as the crystallographic B-factors in such a way that we can compute a model-based map, which can match optimally with the experimental cryo-EM density map (Fig. 1g, h; Supplementary information, Fig. S3 and Data S1).

Resolving water molecules is an important metric for assessing the quality of a true atomic resolution map. We assigned water molecules in our maps using a procedure based on three criteria (Supplementary information, Data S1): a signal to noise threshold to ignore background noise (2-sigma/RMSD above average), the distance between putative water and the closest protein atoms, and the criteria that distinguish water from ions as outlined in reference (Fig. 1i–k).^9^ The distributions show that the procedure places water on well-resolved peaks with Q-scores of 0.5 and higher, even though Q-scores were not used in the selection procedure itself. The radial-distance plot shown in Fig. 1j shows a peak at 2.8 Å between water atoms and nearby O atoms in the protein, as expected.

Recently, two non-peer-reviewed preprints report 1.22 Å and 1.25 Å resolution apoferritin structures^10,11^ using new electron optics (i.e., cold field emission gun, second-generation spherical aberration lens corrector/monochromator), which are aimed to optimize the high-resolution signals^12^ by minimizing the deleterious effects of electron energy spread or lens aberration. In our study, we show two ~1.35 Å resolution structures of the apoferritin without these hardware upgrades. We here selected four representative residue types for a detailed comparison at the individual atom level among these 4 cryo-EM maps, in addition to a 1.01 Å resolution crystal structure,^13^ and a 1.75 Å resolution cryo-EM map (Supplementary information, Fig. S4).^3^ As expected, the two 1.22 Å and 1.25 Å resolution cryo-EM maps have slightly higher Q-scores based on numerical ranking as well as slightly better atomic separations based on visual inspection than our 1.34 Å and 1.36 Å resolution maps.

Another assessment of these cryo-EM structures was to compare the number of water molecules placed in different cryo-EM maps with the criteria described above (Supplementary information, Fig. S5). As expected, more water molecules were found in higher-resolution maps: 216, 217, 170, 165, and 126 waters per protomer in the 1.22 Å, 1.25 Å, 1.34 Å, 1.36 Å, and 1.75 Å resolution maps, respectively (Supplementary information, Fig. S5). Sharper peaks were observed in the distances between the water molecules and the adjacent protein atoms at higher resolution, meaning that higher resolution maps localize water molecules more accurately (Supplementary information, Fig. S5a–e). Molprobity results showed only a few of the water molecules placed with our procedure were found to clash (3 in the 1.34 Å resolution map and 8 in the 1.36 Å resolution map) (Supplementary information, Fig. S2).

When comparing different cryo-EM maps, many water molecules found in one map were also found within 1.0 Å in the other maps (Supplementary information, Fig. S5f). Between our two 1.34 Å and 1.36 Å resolution maps, 72% of the water atoms were within 1.0 Å of each other. We also found that our water placement matches 10% better with the 1.25 Å resolution map (human apoferritin, same as ours) than with the 1.22 Å resolution map (mouse apoferritin), probably due to the difference in species. This is supported by the observation of a bigger difference in the water placement between the 1.22 Å and 1.25 Å resolution maps. Based on these comparisons, it is encouraging to note that a high percentage of water molecules placed in cryo-EM maps from the same species agree, suggesting good reproducibility of water positions across data sets recorded in different electron microscopes with different sample preparations. When comparing our cryo-EM maps to the X-ray structure, a lower percentage (~42%) of water molecules were within the same 1.0 Å distance (Supplementary information, Fig. S5f, g). Given the differences in composition and concentration of the solvent and the chemical environment (protein packing in X-ray crystal vs vitrified single particles in cryo-EM), it is not surprising that there are differences in water positions. Such discrepancy also applies to ion placements (Supplementary information, Data S1). In placed ions, positions compared amongst cryo-EM maps showed lower similarity (~34%) due to different solvent and chemical environments. In comparing our two cryo-EM maps, 11 ions including 7 divalent and 4 monovalent ions were found in equivalent positions within 1.0 Å to each other. However, water and ion identification is still under active research even in atomic resolution X-ray structures,^9^ and it is an emerging and potentially important area in cryo-EM map analysis.

From the overall and detailed evaluation, our 1.34 and 1.36 Å resolution cryo-EM maps show similar characteristic atomic features as the 1.22 Å and 1.25 Å resolution cryo-EM maps and the 1.01 Å resolution crystal structure, and all are certainly better than the 1.75 Å resolution map (Supplementary information, Figs. S4, S5). Such similarity is borne out by the RMSD of all atoms to be around 0.2 Å between ~1.2 Å and ~1.3 Å resolution maps. Nevertheless, the quantitative assessment of the atom resolvability shows a slight improvement of 1.22–1.25 Å over 1.34–1.36 Å resolution cryo-EM maps (Supplementary information, Figs. S4, S5). Our results demonstrate that an atomic resolution structure can be obtained on a 300 kV Titan Krios G3i microscope using the K3 or Falcon 4 detectors with or without an energy filter (Supplementary information, Table S1), which thus gives users having this kind of instrumentation the possibility of performing single-particle cryo-EM analysis at the atomic resolution level. Practically speaking, structures should be sufficient to describe the atom locations in a macromolecular complex equally well in this range of resolution between 1.2–1.3 Å. However, there could still be great interest in the chemical and pharmacological chemistry community to seek more detailed information better than 1.0 Å resolution for understanding basic chemistry and drug design.

Finally, we would like to remind that apoferritin, with its high stability, rigidity, and symmetry, can easily be resolved at atomic resolution by either cryo-EM or X-ray crystallography. However, many biological samples are compositionally or conformationally heterogeneous, as well as often difficult to prepare with the current cryo-freeze plunge method.^14^ These technical hurdles can hinder solving their structures at atomic resolution. Achieving atomic resolution structures is not yet a routine task, but with the further development of cryo-specimen preparation, hardware, and software, it should be possible to apply this approach to an even broader spectrum of macromolecules in the context of chemistry to understand the mechanism and/or to apply it in the drug design pipeline.

## Supplementary information

Supplementary Information

## Data Availability

Cryo-EM maps of the apoferritin structures in this study with their associated atomic models have been deposited in the wwPDB OneDep System under EMD accession codes 22657, 22658 and PDB ID codes 7k3v, 7k3w, respectively.
