# Supplementary material for: Resolving individual atoms of protein complex by cryo-electron microscopy
Source: Cell Res. 2020 Nov 2;30(12):1136–9. doi: 10.1038/s41422-020-00432-2 (PMC7605492; doi:10.1038/s41422-020-00432-2)
Supplement: Supplementary file 1 — Supplementary Information [file 41422_2020_432_MOESM1_ESM.pdf]

Supplementary Information

Resolving Individual Atoms of Protein Complex by Cryo-Electron Microscopy

Kaiming Zhang[a,1], Grigore D. Pintilie[a], Shanshan Li[a], Michael F. Schmid[b], Wah Chiu[a,b,1]

[a]Department of Bioengineering and James H. Clark Center, Stanford University, Stanford, CA 94305, USA

[b]Division of CryoEM and Bioimaging, SSRL, SLAC National Accelerator Laboratory, Menlo Park, CA 94025, USA

[1]Correspondence: Email: wahc@stanford.edu or kmzhang@stanford.edu

Supplementary information, Data S1

Materials and Methods

Cryo-EM sample vitrification and data acquisition

The human ferritin heavy chain sample, in a buffer containing 50 mM Tris-HCl (pH 8.0), 150 mM NaCl was provided by Dr. Xiaojun Huang and Dr. Fei Sun from Institute of Biophysics Chinese Academy of Sciences, China. Three microliters of the samples at 1.5 mg/mL or 0.2 mg/mL concentration were applied onto glow-discharged 200-mesh R2/1 Quantifoil grids (used for the K3 dataset) or 200-mesh R2/1 Quantifoil grids coated with continuous carbon film (used for the Falcon 4 dataset), respectively. The blotting paper was standard Vitrobot filter paper Ø55/20mm, Grade 595 (TED PELLA, INC.). The grids were blotted for 4 s or 2 s and rapidly plunged into liquid ethane using a Vitrobot Mark IV (Thermo Fisher Scientific) at 4°C and 100% humidity. The first dataset (K3 dataset) was imaged in a Titan Krios cryo-electron microscope (Thermo Fisher Scientific) operated at 300 kV with GIF energy filter (Gatan) at a magnification of 215,000× (corresponding to a calibrated sampling of 0.4 Å per pixel). Micrographs were recorded by EPU software (Thermo Fisher Scientific) with a Gatan K3 Summit direct electron detector, where each image was composed of 30 individual frames with an exposure time of 0.5 s and an exposure rate of 90 electrons per second per $Å^2$. A total of 8,034 movie stacks were collected. The second dataset (Falcon 4 dataset) was imaged in another Titan Krios cryo-electron microscope (Thermo Fisher Scientific) operated at 300 kV at a magnification of 155,000× (corresponding to a calibrated sampling of 0.502 Å per pixel). Micrographs were recorded by EPU software (Thermo Fisher Scientific) with a Falcon 4 direct electron detector, where each image was composed of 40 individual frames with an exposure time of 2 s and an exposure rate of 20 electrons per second per $Å^2$. A total of 7,734 movie stacks were collected.

Single-particle image processing and 3D reconstruction

All micrographs were first imported into Relion for image processing. The motion-correction was performed using Relion's own implementation and the contrast transfer function (CTF) was determined using CTFFIND4[1]. For the K3 dataset, 6,951 micrographs were selected with a defocus

range from -0.35 to -1.3 μm, and “rlnCtfMaxResolution < 4.5”. For the Falcon 4 dataset, 5,427 micrographs were selected with a defocus range from -0.3 to -1.3 μm, and “rlnCtfMaxResolution < 4”. All particles were autopicked using the NeuralNet option in EMAN2[2]. Then, particle coordinates were imported to Relion, where the poor 2D class averages were removed by two rounds of 2D classification. The initial models for both datasets were built in cryoSPARC[3] using the ab-initio reconstruction option with octahedral symmetry applied. For the K3 dataset, 1,176,336 particles were picked and 902,455 were selected after 2D classification. For the Falcon 4 dataset, 707,350 particles were picked and 500,643 were selected after 2D classification. The 3D refinement was performed using the particle images selected from 2D classification with further “CTF refinement and Bayesian polishing” in Relion. A 1.34 Å resolution map from the K3 dataset and a 1.36 Å resolution map from the Falcon 4 dataset were obtained (Supplementary information, Fig. S1). Resolutions of the final maps were estimated with the 0.143 criterion of the Fourier shell correlation curve. The figures were prepared using UCSF Chimera[4] .

Model Fitting and Refinement

Segger (v.2.5) was used to first segment the 1.34 Å resolution cryo-EM map into regions corresponding each of the 24 protein subunits (using Group by Connectivity, 30 steps, at a threshold of 0.01)[5]. The X-ray structure of human apoferritin (PDB:3ajo[6]) was fitted to one of the segments using the Fit to Segments dialog. The structure was then refined using phenix.real_space_refine[7]. It was visually inspected in Chimera[4] residue by residue to ensure a proper fit. No problems were seen in the backbone. Some side chains however did not fit into the observed density and were re-modeled to fit properly using the Rotamers dialog in Chimera. For several residues the map showed more than one possible rotamer, and alternate conformations were added using the same dialog. The resulting structure was then refined one more time with phenix.real_space_refine to allow the re-modeled side chains to be adjusted further into the density. The same process was performed for the 1.36 Å resolution map, segmenting at a threshold of 0.03 (also with 30 steps of grouping by connectivity), and fitting the model refined into the 1.34 Å resolution map. On visual inspection, all residues appeared to fit very well to the map and no manual adjustments were needed. The phenix.real_space_refine procedure however was applied and the model changed slightly into the 1.36 Å resolution structure.

Q-score adjustment

Q-scores are calculated by correlating map values around each atom to a "reference Gaussian". In our previous paper describing the Q-score[8], the width (sigma) of the reference Gaussian was set to 0.6 Å, which resulted in the maximum Q-score of ~1.0 at a resolution of 1.5 Å. Using this definition, Q-scores start to drop at resolutions higher than 1.5 Å, as atom peaks become sharper than the reference Gaussian. Hence, we adjusted sigma to 0.4 Å, so that Q-scores are now highest at a resolution of ~1.1 Å, and a linear correlation can again be seen between Q-scores and resolutions (Supplementary information, Fig. S6). Smaller sigma values will again be required if resolutions continue to increase past 1.1Å.

B' calculations

We calculate B' factors from atom Q-scores using the following empirically derived formula:

$$B' = f*(1-Q)$$

This formulation establishes the relationship that atoms with higher Q-scores produce lower B'-factors, as they are better resolved. We determined the best scaling factor, *f*, by trying several values (0, 50, 100, 200, 300, 400), and observing which value caused the largest increase in the FSC. Since Q-scores correlate to resolution, whereas B-factors do not, as shown previously[8]. We expect that a different factor *f* will be required at different resolutions.

Segmentation-guided Water and Ion Modeling (SWIM)

To search for water molecules and ions, we used conceptual criteria described in reference[9] as summarized below:

- A "placed water" that clashes (i.e. is closer than the typical hydrogen bond distance) with two or more atoms of the same polarity, and with no non-polars (C) or opposite polars (O and N), is almost certainly an ion.
- If the "placed water" clashes (is too close) to negative atoms, it is a positive ion.
- If the 'placed water' clashes with positive atoms, it is a negative ion.
- A doubly charged ion (e.g. Mg^{2+}, Fe^{2+} or Zn^{2+}) almost always interacts with at least one fully charged atom (e.g. phosphate or carboxyl O).
- A singly charged ion (e.g. Na^{+}) often interacts with just partial charges (e.g. OH and backbone CO).

The above criteria[9] did not describe exact distances. We chose the distance ranges used in our procedure based on observed distances between waters/ions and nearby atoms in high-resolution apoferritin crystal structure (PDB:3ajo[6]). They were found as follows:

- Water atom to nearby polar atoms: 2.8 ± ~0.3 Å.
- Ion to nearby charged/polar atoms: 2.2 ± ~0.3 Å.

Based on these criteria and observations, we implement the following procedures. The cryo-EM map is first segmented using the watershed method[10], which produces regions corresponding to peaks in the map. The boundaries between these regions are the lowest values in the map between these peaks. This is basically a peak-finding algorithm. A threshold of 2-sigma above the mean density value in the map is used here, so that the detected peaks are more likely to correspond to signal rather than noise. The resulting regions are then sorted by volume (number of voxels in the region), and considered in decreasing order,

For each region, take the point in it with the highest map density value as its position (P). Then, for each nearby atom to P:

a. If the atom is non-polar and non-charged (e.g. carbon atom) and is within 2.6 Å of P, P is ignored and the search continues with the next regions.
b. If the atom can have a charge in an appropriate local chemical environment (e.g. O in Glutamic acid (Glu)/Aspartic acid (Asp) or N in Lysine (Lys)/Arginine (Arg)/Histidine (His) side chains) and:
    i. If the atom is within a distance of 1.9 Å to 2.5 Å to P, it is added to ChargedAtoms list.
    ii. If the atom is within 2.5 Å to 3.1 Å to P, it is added to WaterAtoms list.
c. If the atom is polar, e.g. O in the backbone, O or N in the side chains that are not typically charged at the experimental pH, and S in Cysteine (Cys), and:
    i. If the atom is within 1.9 Å to 2.5 Å to P, it is added to PolarIonAtoms list (this distance range is characteristic of an ion).
    ii. If the atom is within 2.5 Å to 3.1 Å to P, it is added to PolarWaterAtoms list (this distance range is characteristic of a water molecule).
d. If the ChargedAtoms list is not empty, P is added as a 2+ ion (e.g. Fe^{2+}, Mg^{2+}, Zn^{2+}).

e. Otherwise, if the PolarIonAtoms list is not empty, P is added as a singly-charged ion (e.g. Na^+ if close to O atom or Cl^- if close to N atom).
f. Otherwise, if the PolarWaterAtoms list is not empty, P is added as a water.

To determine what type of double-charged ion (such as Mg^{2+}, Zn^{2+}, Ca^{2+}, Fe^{2+}, and Cu^{2+}) to place may not be directly possible from the density, although some efforts have been made in this direction with X-ray data[11]. The decision of types of ions can be made based on other information such as the buffer condition and knowledge of the protein biochemistry with respect to the metal binding if available.

The procedure above has been integrated in the Segger plugin to UCSF Chimera (v 2.5 and later). The plugin, code, installation, and running instructions are detailed at the github page[12].

References

1. Rohou, A. & Grigorieff, N. CTFFIND4: Fast and accurate defocus estimation from electron micrographs. *J. Struct. Biol.* **192**, 216–221 (2015).
2. Bell, J. M., Chen, M., Baldwin, P. R. & Ludtke, S. J. High resolution single particle refinement in EMAN2.1. *Methods* **100**, 25–34 (2016).
3. Punjani, A., Rubinstein, J. L., Fleet, D. J. & Brubaker, M. A. cryoSPARC: algorithms for rapid unsupervised cryo-EM structure determination. *Nat. Methods* **14**, 290–296 (2017).
4. Pettersen, E. F. *et al.* UCSF Chimera--a visualization system for exploratory research and analysis. *J. Comput. Chem.* **25**, 1605–1612 (2004).
5. Pintilie, G. D., Zhang, J., Goddard, T. D., Chiu, W. & Gossard, D. C. Quantitative analysis of cryo-EM density map segmentation by watershed and scale-space filtering, and fitting of structures by alignment to regions. *J. Struct. Biol.* **170**, 427–438 (2010).
6. Masuda, T., Goto, F., Yoshihara, T. & Mikami, B. The universal mechanism for iron translocation to the ferroxidase site in ferritin, which is mediated by the well conserved

transit site. *Biochem. Biophys. Res. Commun.* **400**, 94–99 (2010).

7. Afonine, P. V. *et al.* New tools for the analysis and validation of cryo-EM maps and atomic models. *Acta Crystallogr D Struct Biol* **74**, 814–840 (2018).

8. Pintilie, G. *et al.* Measurement of atom resolvability in cryo-EM maps with Q-scores. *Nat. Methods* **17**, 328–334 (2020).

9. Prisant, M. G., Williams, C. J., Chen, V. B., Richardson, J. S. & Richardson, D. C. New tools in MolProbity validation: CaBLAM for CryoEM backbone, UnDowser to rethink 'waters,' and NGL Viewer to recapture online 3D graphics. *Protein Sci.* **29**, 315–329 (2020).

10. Beucher, S. and C. Lantuéjoul. Use of watersheds in contour detection. *Proc. Int. Workshop on Image Processing,* Sept. 1979, 17-21 (1979).

11. Echols, N. *et al.* Automated identification of elemental ions in macromolecular crystal structures. *Acta Crystallogr. D Biol. Crystallogr.* **70**, 1104–1114 (2014).

12. Website. Website. Segger v2.5. https://github.com/gregdp/segger.

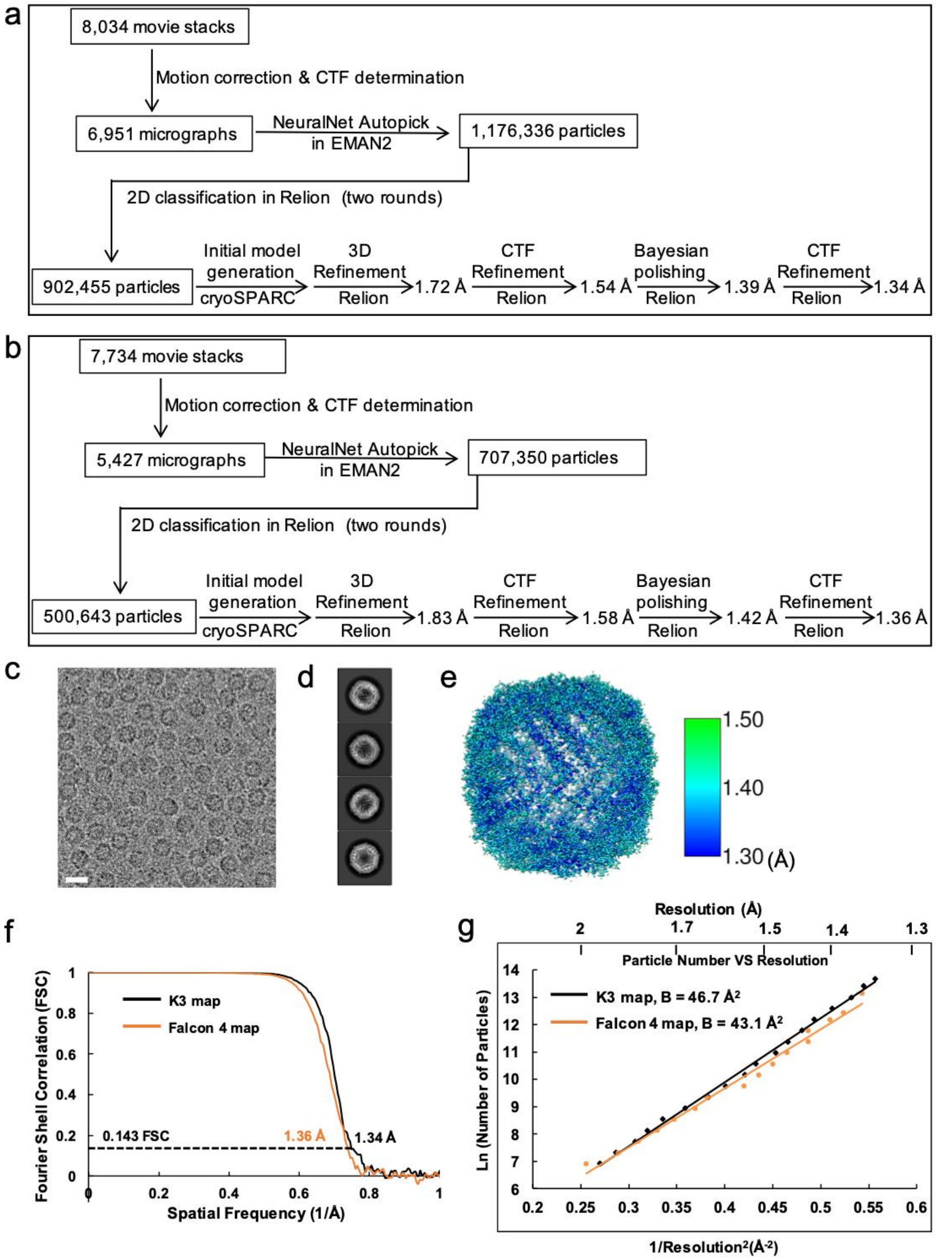

Supplementary information, Fig. S1: Single-particle cryo-EM analysis of apoferritin structures at atomic resolution from two datasets collected on K3 and Falcon 4 detectors. a Image processing workflow of the dataset collected on K3 detector. **b-e** Data from Falcon 4 dataset. **b** Image processing workflow of the dataset collected on Falcon 4 detector. **c** Representative motion-corrected cryo-EM micrograph. The scale bar represents 200 Å. **d** Reference-free 2D class averages of computationally extracted particles. **e** Resolution variation maps for the final 3D reconstruction. **f** Gold standard FSC plots for the final 3D reconstructions for the two maps. **g** Plots

of the particle number vs the reciprocal squared resolution. The B-factor was calculated as 2x the linear fitting slope.

a 1.34 Å resolution K3 Map

All-Atom Contacts	Clashscore, all atoms:	0.68		99th percentile* (N=1784, all resolutions)
	Clashscore is the number of serious steric overlaps (> 0.4 Å) per 1000 atoms.			
Protein Geometry	Poor rotamers	0	0.00%	Goal: <0.3%
	Favored rotamers	162	96.43%	Goal: >98%
	Ramachandran outliers	0	0.00%	Goal: <0.05%
	Ramachandran favored	168	98.82%	Goal: >98%
	Rama distribution Z-score	3.51 ± 0.63		Goal: abs(Z score) < 2
	MolProbity score^	0.72		100th percentile* (N=27675, 0Å - 99Å)
	Cβ deviations >0.25Å	0	0.00%	Goal: 0
	Bad bonds:	0 / 1522	0.00%	Goal: 0%
	Bad angles:	1 / 2049	0.05%	Goal: <0.1%
Peptide Omegas	Cis Prolines:	1 / 3	33.33%	Expected: ≤1 per chain, or ≤5%
Low-resolution Criteria	CaBLAM outliers	1	0.6%	Goal: <1.0%
	CA Geometry outliers	0	0.00%	Goal: <0.5%
Additional validations	Chiral volume outliers	0/219		
	Waters with clashes	0/161	0.00%	See UnDowser table for details

b

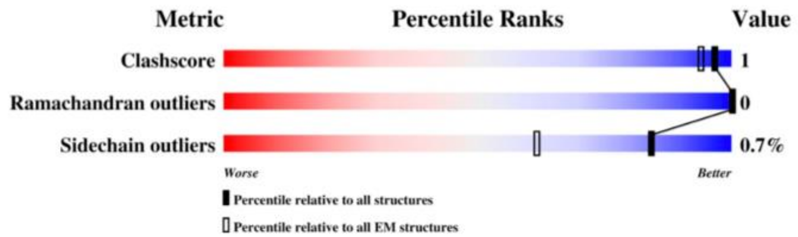

c 1.36 Å resolution Falcon 4 Map

All-Atom Contacts	Clashscore, all atoms:	0.68		99th percentile* (N=1784, all resolutions)
	Clashscore is the number of serious steric overlaps (> 0.4 Å) per 1000 atoms.			
Protein Geometry	Poor rotamers	0	0.00%	Goal: <0.3%
	Favored rotamers	161	96.41%	Goal: >98%
	Ramachandran outliers	0	0.00%	Goal: <0.05%
	Ramachandran favored	167	98.24%	Goal: >98%
	Rama distribution Z-score	3.36 ± 0.63		Goal: abs(Z score) < 2
	MolProbity score^	0.76		100th percentile* (N=27675, 0Å - 99Å)
	Cβ deviations >0.25Å	0	0.00%	Goal: 0
	Bad bonds:	0 / 1517	0.00%	Goal: 0%
	Bad angles:	0 / 2042	0.00%	Goal: <0.1%
Peptide Omegas	Cis Prolines:	1 / 3	33.33%	Expected: ≤1 per chain, or ≤5%
Low-resolution Criteria	CaBLAM outliers	1	0.6%	Goal: <1.0%
	CA Geometry outliers	0	0.00%	Goal: <0.5%
Additional validations	Chiral volume outliers	0/218		
	Waters with clashes	0/163	0.00%	See UnDowser table for details

d

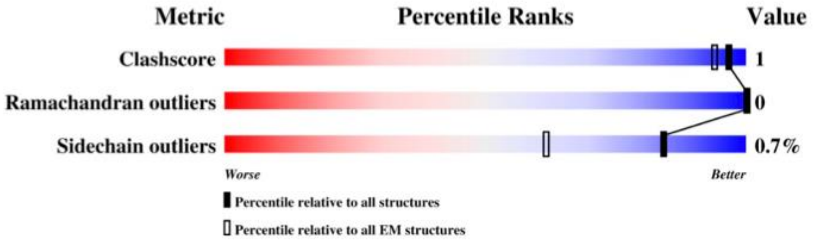

Supplementary information, Fig. S2: Assessment of model quality. MolProbity analysis (**a, c**) and overall quality derived from PDB validation reports (**b, d**) for atomic models fitted to 1.34 Å (**a, b**) and 1.36 Å (**c, d**) resolution maps. Both reports show good model geometries and very few water atoms that clash with other atoms.

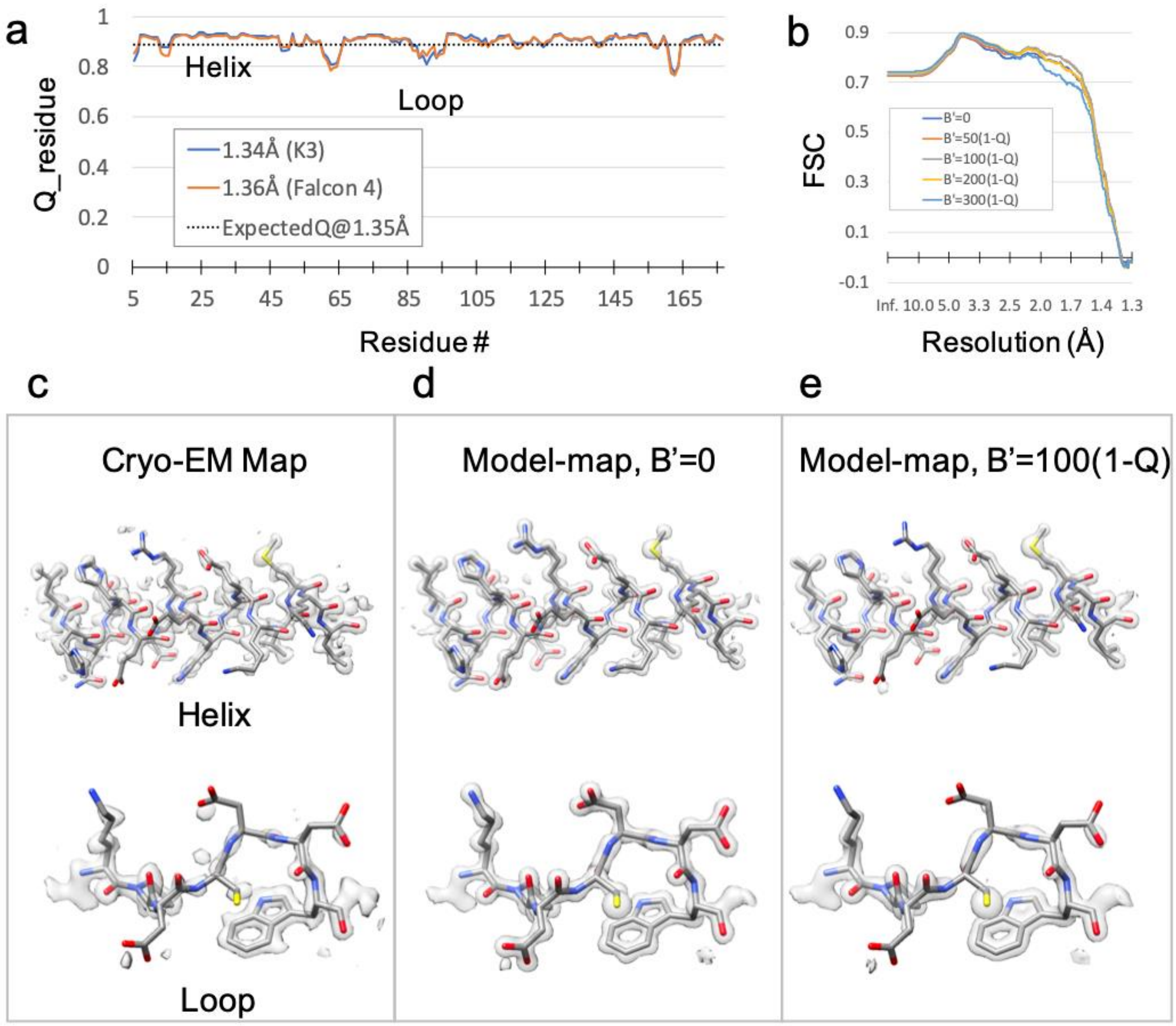

Supplementary information, Fig. S3: Q-scores and B' factors. a Plots of per-residue Q-score for 1.34 Å and 1.36 Å resolution maps. Most residues have Q-scores above the expected Q-score for a resolution of 1.34 Å, with just a few dips which occur mostly in loop regions. **b** FSC plots between the 1.34 Å resolution map and model are shown, using several B' scaling factors (see Methods). The scaling factor of B'=100(1-Q) has the highest FSC correlations at all resolutions. **c-e** Two extracted helical and loop regions to show the consistency between cryo-EM map and model-derived map. In (**c**), the cryo-EM map is shown. In (**d**), a model-generated map with B'-factors of 0 is shown; all atoms are inside the contour shown, unlike in the cryo-EM map. In (**e**), a model-generated map with B'-factors calculated from Q-scores is shown; here, atoms that have lower resolvability in the cryo-EM map are similarly un-resolved in the model-generated map.

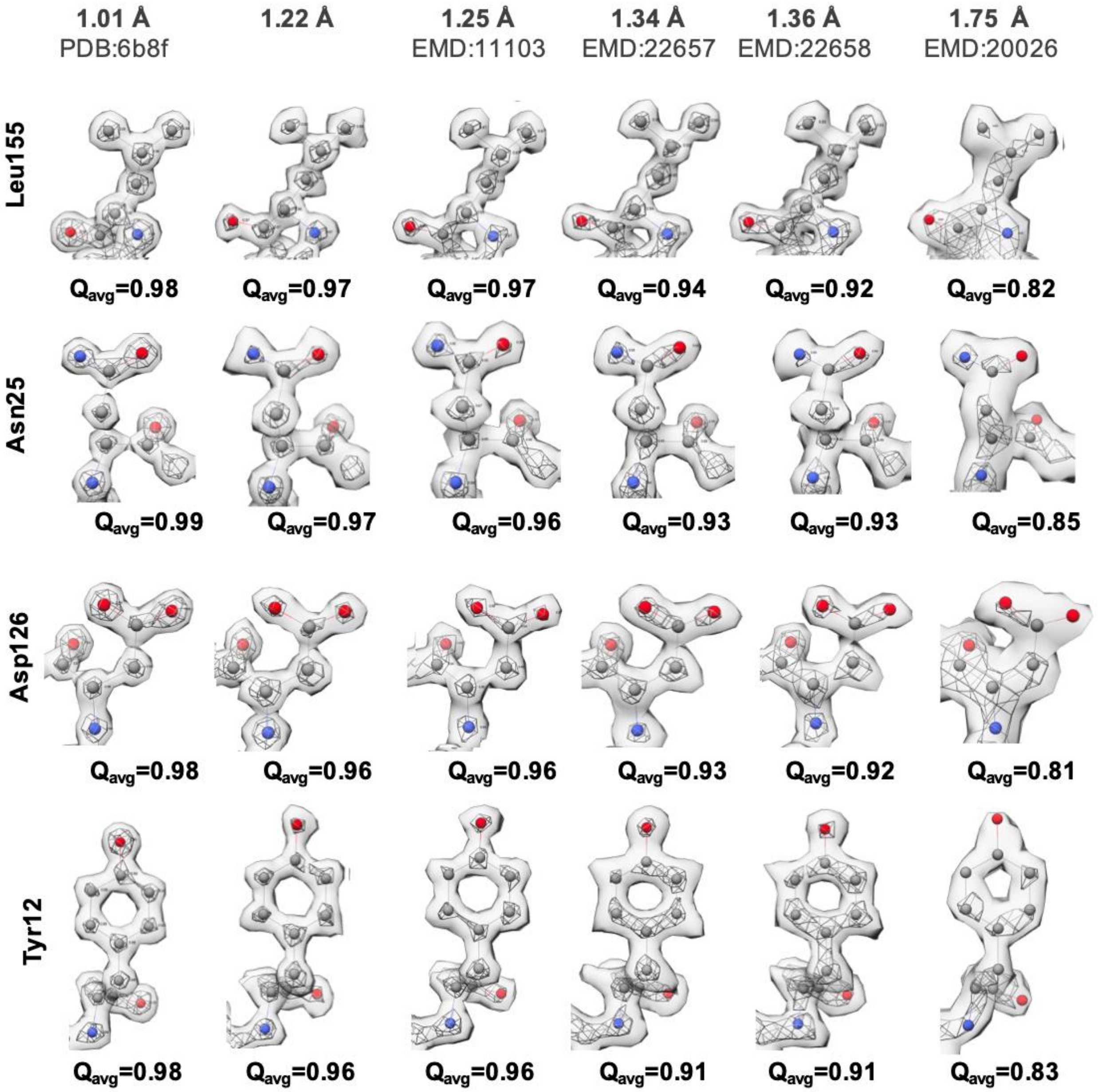

Supplementary information, Fig. S4: Comparing the resolvability of apoferritin structures at multiple resolutions by Q-score. Four different residues were selected based on the type of side chain (polar, charged, and hydrophobic). The residues are shown by element (grey, carbon; red, oxygen; blue, nitrogen). The 1.22 Å resolution map was downloaded from Scheres lab (ftp://ftp.mrc-lmb.cam.ac.uk/pub/scheres/atomic/).

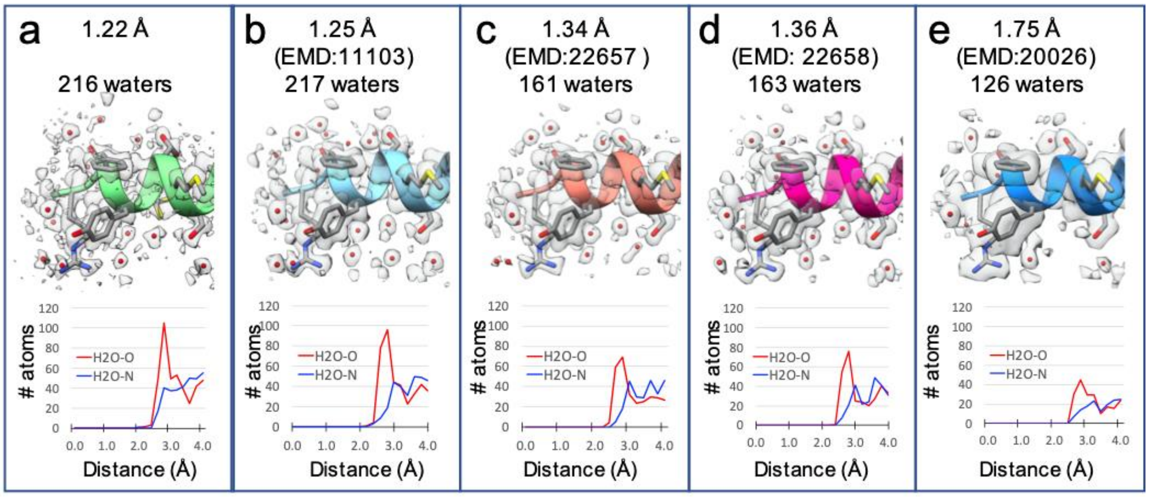

f

	# water	1.22 Å	1.25 Å	1.34 Å	1.36 Å	1.75 Å
1.22 Å	216	-				
1.25 Å (EMD:11103)	217	123 (57%)	-			
1.34 Å (EMD:22657)	170	105 (62%)	124 (73%)	-		
1.36 Å (EMD:22658)	165	112 (68%)	120 (73%)	118 (72%)	-	
1.75 Å (EMD:20026)	126	81 (64%)	77 (61%)	71 (56%)	71 (56%)	-
PDB:3ajo	229	95 (41%)	109 (48%)	93 (41%)	99 (43%)	60 (26%)

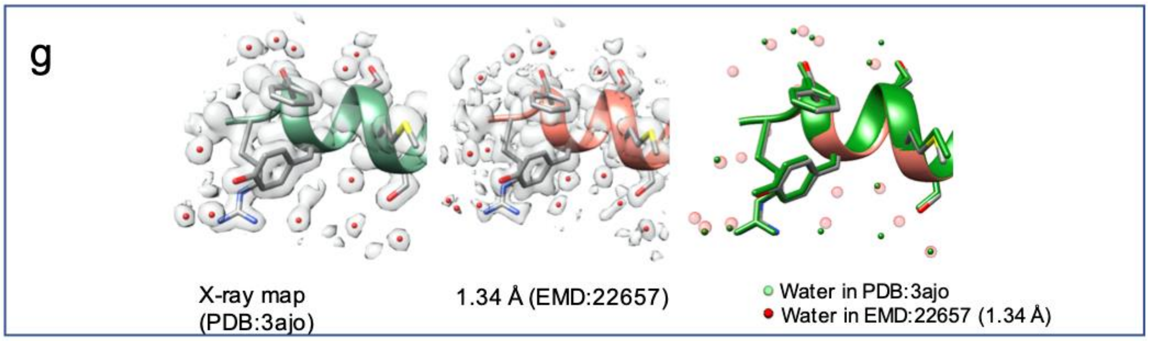

Supplementary information, Fig. S5: Water assignment in apoferritin maps at different resolutions. a-e Extracted regions from five cryo-EM maps show water molecules near protein atoms, along with radial-distance plots. The latter shows peaks at a distance of 2.8 Å from protein O atoms. **f** Comparison of water molecules placed in different cryo-EM maps. The first column shows the number of water molecules found in each map. The other columns are N × N comparisons of the number of waters that are within 1.0 Å of each other in two different maps. **g** Comparison of water molecules between an X-ray structure (PDB: 3ajo) and our 1.34 Å resolution map. The 1.22 Å resolution map was downloaded from Scheres lab (ftp://ftp.mrc-lmb.cam.ac.uk/pub/scheres/atomic/).

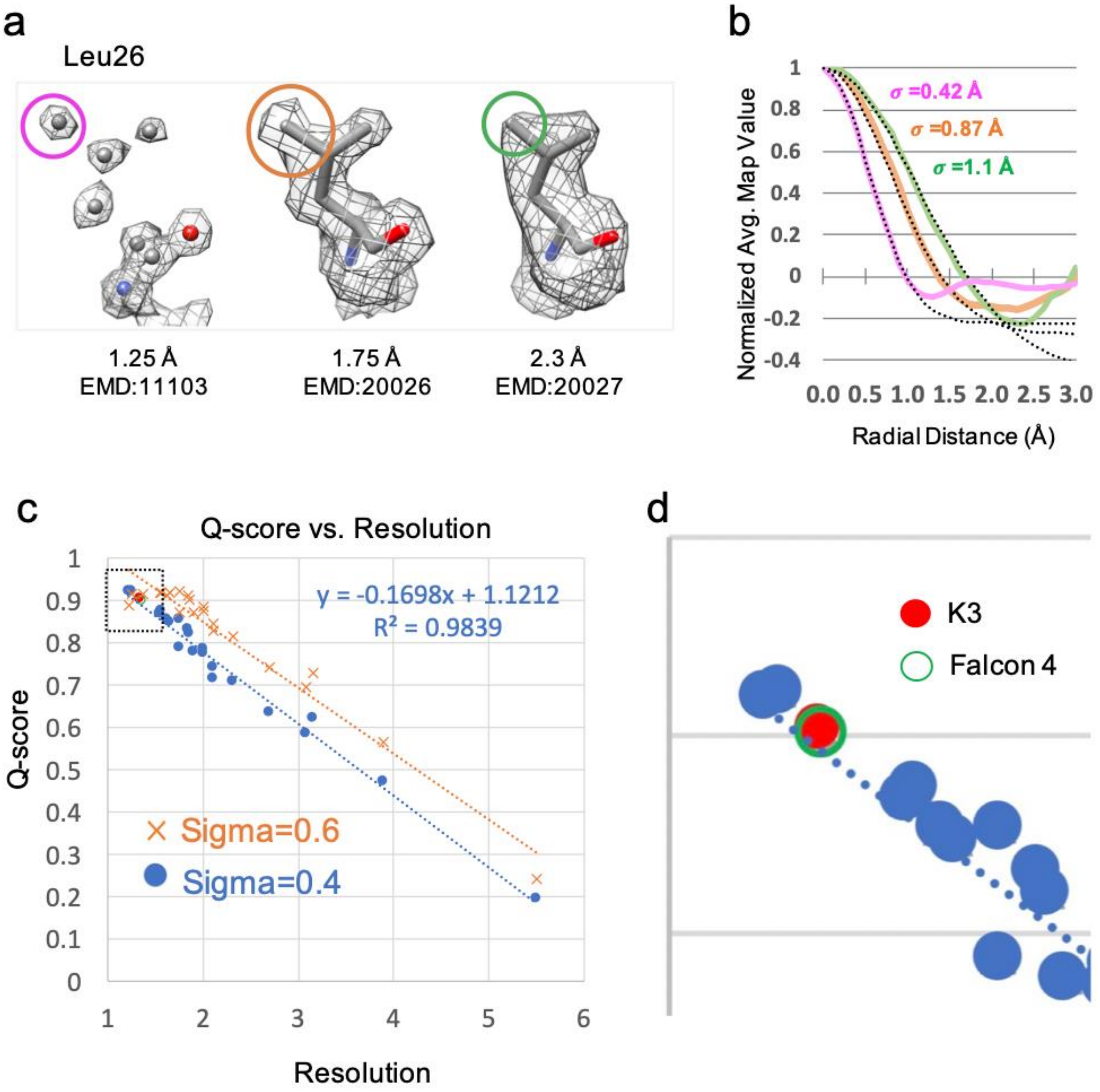

Supplementary information, Fig. S6: Q-score adjustment for apoferritin maps at better than 1.5 Å resolution. Atoms in maps at 1.25 Å, 1.75 Å, and 2.3 Å resolution (**a**) all have very close Gaussian-like atomic profiles with smaller widths (***σ***) at higher resolutions (**b**). Previously, Q-scores were calculated using a sigma parameter of 0.6, which gave the highest Q-score of 1 at a resolution of ~1.5 Å. With this parameter, Q-scores start to drop at higher resolutions as shown in (**c**). Thus, we adjusted the Q-score calculation to give the highest score of 1.0 at ~1.2 Å by setting sigma to 0.4; with this parameter, the plot of Q-score vs. resolution for 10 maps & models in the EMDB is now linear again. Panel (**d**) shows a close-up of (**c**) at resolutions between 1-2 Å, highlighting the Q-scores for the new maps of apoferritin at 1.34 Å (K3) and 1.36 Å (Falcon 4) resolution.

Supplementary information, Table S1. Cryo-EM data collection and processing

Apoferritin	K3 dataset	Falcon 4 dataset
**Data collection and processing**		
Microscope	Titan Krios G3i	Titan Krios G3i
Voltage (kV)	300	300
Camera	Gatan K3	Thermo Fisher Falcon 4
Grids Type	R2/1 Quantifoil grids	R2/1 Quantifoil grids coated with continuous carbon film
Sample concentration	1.5 mg/mL	0.2 mg/mL
Magnification	215,000x	155,000x
C2 aperture size (µm)	70	70
Objective aperture size (µm)	No	No
Pixel size (Å)	0.4	0.502
Total exposure (e-/Å^2)	45	40
Exposure time (s)	0.5	2
Number of frames per exposure	30	40
Energy filter slit width (eV)	15	No
Data collection software	EPU 2.7	EPU 2.7
Maximum image shift (µm)	12	12
Number of exposures per hole	6	8
Defocus range (µm)	-0.35 to -1.3	-0.3 to -1.3
Number of micrographs collected	8,034	7,734
Number of micrographs used	6,951	5,427
Number of initial particles	1,176,336	707,350
Symmetry	Octahedral	Octahedral
Number of final particles	902,455	500,643
Resolution (0.143 gold standard FSC, Å)	1.34	1.36
Local resolution range (Å)	1.3 - 1.5	1.3 - 1.5
